# Cutaneous, Cranial, and Skeletal Defects in Children and Adults with Focal Dermal Hypoplasia

**DOI:** 10.3390/children10101715

**Published:** 2023-10-22

**Authors:** Ali Al Kaissi, Sergey Ryabykh, Vladimir Kenis, Farid Ben Chehida, Hamza Al Kaissi, Susanne Gerit Kircher, Franz Grill

**Affiliations:** 1 National Medical Research Center for Traumatology and Orthopedics, 640014 Kurgan, Russia; 2 Veltischev Clinical Institute, Pirogov Russian National Research Medical University, 117997 Moscow, Russia; rso_@mail.ru; 3 Department of Foot and Ankle Surgery, Neuroorthopaedics and Systemic Disorders, Pediatric Orthopedic Institute, Parkovaya Str., 64-68, Pushkin, 196605 Saint Petersburg, Russia; kenis@mail.ru; 4 Ibn Zohr Institute of Diagnostic Radiology, Cite Al Khadra, Tunis 1003, Tunisia; if.chehida@gnet.tn; 5 Clinic for Dermatology and Allergology, Luisen Hospital, 52064 Aachen, Germany; alkaissihamza@gmail.com; 6 Center of Medical Patho-Biochemistry and Genetics, Medical University of Vienna, 1090 Vienna, Austria; sus@skirch.at; 7 Pediatric Department, Orthopedic Hospital of Speising, 1130 Vienna, Austria; grill.franzleo@gmail.com

**Keywords:** focal dermal hypoplasia, Goltz syndrome, defective cranial and spine ossifications, split hand/foot (Ectrodactyly), CT scan

## Abstract

Background: The diagnostic process for children and adults manifesting a constellation of ectodermal abnormalities requires a conscientious and highly structured process. Material and Methods: Six girls (aged 6-month–8 years) and two older girls (aged 13 and 16 years) were born with variable skin lesions of varying intensities associated with noticeable cranial and skeletal malformation complexes. Cleft palate, abnormal dentition, and multiple papillomas were evident around the mouth, mostly bilateral but asymmetrical in the upper and lower limbs. Exaggerated frontal bossing (macrocephaly) and in some patients’ microcephaly with variable skeletal defects of the craniocervical junction and diverse forms of lower limb deformities of syndactyly, polydactyly, and split-hand/foot (ectrodactyly). Results: All patients manifested the constellation of abnormalities with variable intensities ranging between alopecia, papillomas, striated skin pigmentations split-hand/foot (ectrodactyly), and major bone defects. A 3D reconstruction CT scan was directed mainly to further scrutinize children with pseudo cleft lip, submucus cleft, and cleft palate. Interstingly, they manifested massive demineralization of the cranium associated with severely defective dentition. A spine 3D reconstruction CT scan in two girls showed marked cystic cavitation of the upper jaw associated with excessive cavitation of the mastoid, causing tremendous frailty of the mastoid bone. A 3D sagittal CT scan showed odontoid hypoplasia and C1-2 instability associated with the rudimentary atlas and the persistence of extensive synchondrosis of the cervico-thoracic spine. The overall clinical and radiological phenotypic characterizations were consistent with the diagnosis of focal dermal hypoplasia (Goltz syndrome). Two children manifested heterozygous mutations in the PORCN gene, chromosome Xp11. Conclusions: In this study, we believe it’s a good opportunity to share our novel scientific findings, which are intriguing and can be inspiring to readers, and to further aid the current scientific literature with exceptionally new unveiling results. This is the first comprehensive study of the cranio-skeletal malformation complex in children with GS.

## 1. Introduction

The skin lesions in patients with focal dermal hypoplasia (Goltz syndrome) are characteristic (OMIM#305600). Focal dermal hypoplasia is inherited in an X-linked dominant pattern. It is well known that it is a lethal syndrome in males. The features include atrophy and linear pigmentation of the skin, herniation, and multiple papillomas around the mouth and in the mucous membranes or skin. In addition, upper and lower limb anomalies consist of a variable malformation complex. Oral anomalies, in addition to lip papillomas, include hypoplastic teeth, a cleft lip and palate, and abnormal dentition [1,2,3]. GS ranges between congenital skin hypoplasia, which might be severe and spread over the scalp, alopecia, and anophthalmos. In most cases, the skin stigmata are usually bilateral but asymmetrical. Skin stigmata are initially red in color and patchy, alternating with areas of linear or reticular hyper- or hypopigmentation. Papillomas are mostly seen around the lips, gums, or the side of the nose [4]. The limb defects are usually asymmetrical, including syndactyly, postaxial polydactyly, hypoplastic phalanges, bifid thumb, split/cleft foot/ectrodactyly [5].

The vast majority of the patients are female, and inheritance is thought to be X-linked dominant with an early history of stillbirths. In the literature, there have been some papers denoting father-to-daughter transmission [5]. *PORCN* has been encountered in patients with Goltz syndrome (focal dermal hypoplasia) as the causative gene. The *PORCN* gene is usually located on the X chromosome, and Goltz syndrome is inherited through an X-linked dominant pattern. The X-linked dominant disorders are due to abnormal genes on the X chromosome and occur mostly in females [6,7]. The split hand/split foot deformity can occur in patients with ectodermal dysplasia and particularly in EEC syndrome (ectrodactyly, ectodertmal dysplasia, and clefting); this is in comparison to the X-linked type of inheritance, also seen in autosomal dominant and recessive types [8,9]. In our current families, spontaneous abortions in the first trimester and stillbirths of male infants might explain the extreme expression of the disordered gene. In short, this study signifies the necessity to explore the cranium and the skeletal system in patients with Goltz syndrome. The optimal outcome for the management of these patients can be better achieved when we understand the depth of the associated pathologies. The treatment plan for split hand/foot deformities, coupled with the existence of cleft palate and craniocervical pathologies, made any surgical intervention quite difficult to achieve success.

## 2. Material and Methods

The study protocol was approved by the Ethics Committee of the (Ilizarov Scientific Research Institute, No. 4(50)/13.12.2016, Kurgan, Russia). Informed consents were obtained from the patient’s guardians. Six girls (aged 6-month–8 year) and two older girls (aged 13 and 16 years) were born with variable, though distinctive, skin lesions of varying intensities associated with noticeable cranial and skeletal malformation complexes. All were referred to our departments because of dysmorphic craniofacial, dermatological, and limb defects. They showed growth deficiency (10–25th percentile). Craniofacial features of frontal bossing, bifid frontal area, alopecia areata, hypotrichosis (sparse hair), faint eyebrows/eyelashes, microphthalmia, deep-seated eyes, and strabismus. Severely dysplastic, low-set, and apparently outstanding bulging ear (mostly unilateral) associated with pits over the tragus, microstomia/macrostomia with hypertrophied gums, dysplastic oligodontia, high vault palatine, cleft palate, and in three other girls, submucous clefting, wart-like papilloma over the upper lip, around the mouth, and in the mucous membranes. Extensive dissemination of hypopigmented/hpoplastic skin spots over the chest and hypoplasia of the nipples and the upper and lower limbs. We literally describe the evolution of the disease from infancy up to pre-adolescence. The children have had skin lesions since birth. Started as linear vesicular eruptions, which can be seen on the trunk as well as the upper and lower limbs. The linear form is followed by warty verrucous eruptions, which persisted for a long time and were modified by the appearance of transverse or whorled hyperpigmentation with spots of hypopigmentation all over the body. Special attention is given to children with pseudo-clefts, submucus clefts, and cleft palates. From our experience, we strongly believe in the hazardous maldevelopment of the craniocervical junction (specifically the atlas) in children with clefting syndromes. Therefore, these children underwent profound tomographic studies to confirm or rule out the extension of the clefting process. Omitting the clefting pathology over the craniocervical junction can lead to a series of unpleasant neurological deficits associated with serious vertibrobasilar ischemia.

Intellectual disability has been encountered in two patients. Clinical and radiological phenotypic characterization are our first steps towards the fulfillment of the diagnostic process. Prior to any surgical arrangements, especially reconstruction surgical interventions. We diagnosed GS patients in accordance with the detection of major criteria and minor criteria. The examination of siblings and parents has been performed with maximal care following the aforementioned strategy. One family showed a mother who manifested alopecia, papillomas, oligodontia, enamel dysplasia, and syndactyly. All candidates for operations underwent extensive cranio-cervical junction study via radiological and tomographic examinations, especially for the cranium, craniocervical, and spine, to guard against unpleasant or tragic events that might lead to morbid or mortal complications. Patients with Goltz syndrome are heterozygous or mosaic for a pathogenic variant in the *PORCN gene mutation.* We sub-categorized our patients in accordance with the most prominent cutaneous, cranial, as well as other skeletal malformations.

These abnormalities can result in neck pain, syringomyelia, cerebellar, lower cranial nerve, and spinal cord deficits, and vertebrobasilar ischemia.

## 3. Results

### 3.1. Cranio-Facial Features of Goltz Syndrome

The craniofacial features in children and adults with multiple malformation complexes, as in GS, are the cornerstone from which we begin to start our diagnostic process. The unusual craniofacial features, through the appearance of distinctive dysmorphic criteria, are not always properly read by pediatricians, particularly when it comes to minor malformations; these can readily be omitted. We describe three unrelated children with GS by focusing on the major and minor craniofacial changes.

**Figure 1:**
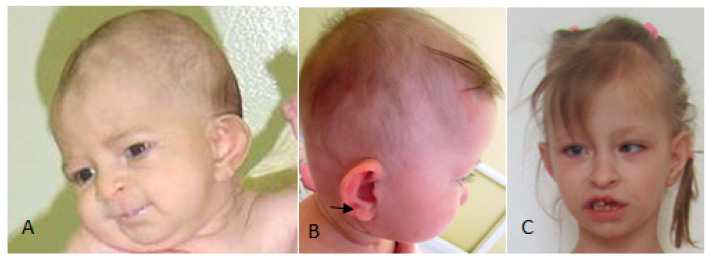
Cranio-facial features in a 6-month-old girl with GS showed sparse hair, frontal bossing, sloping of the cranial vault, bilateral bossing of the supra-zygomatic arches, sparse eye lashes with no eyebrows, deeply seated eyes, a unilateral dysplastic low-set ear with incomplete folding, a depressed nasal root, notching of the sides of the nose, and mouth puckering (**A**). A lateral view of a 20-month-old child with GS showed sparse hair, frontal bossing, large protruding/dysplastic, and a low-set ear associated with pitting over the tragus (**B**). Facial features of an 8-year-old girl with GS showed sparse hair, frontal bossing, faint eyebrows, strabismus, craniofacial asymmetry, unilateral dysplastic left ear, notching of the sides of the nose, short philtrum, macrostomia, and wart-like papilloma around and over the upper lip, oligodontia, and high vault palatine (**C**).

Cranio-facial features in a 6-month-old girl with GS showed sparse hair, frontal bossing, sloping of the cranial vault, bilateral bossing of the supra-zygomatic arches, sparse eye lashes with no eyebrows, deeply seated eyes, a unilateral dysplastic low-set ear with incomplete folding, a depressed nasal root, notching of the sides of the nose, and mouth puckering (Figure 1A). A lateral view of a 20-month-old child with GS showed sparse hair, frontal bossing, large protruding/dysplastic, and a low-set ear associated with pitting over the tragus arrow (Figure 1B). The facial features of an 8-year-old girl with GS showed sparse hair, frontal bossing, faint eyebrows, strabismus, craniofacial asymmetry, unilateral dysplastic left ear, notching of the sides of the nose, short philtrum, macrostomia, and wart-like papilloma around and over the upper lip, oligodontia, and high vault palatine (Figure 1C).

### 3.2. Skin Phenotype in Goltz Syndrome

**Figure 2:**
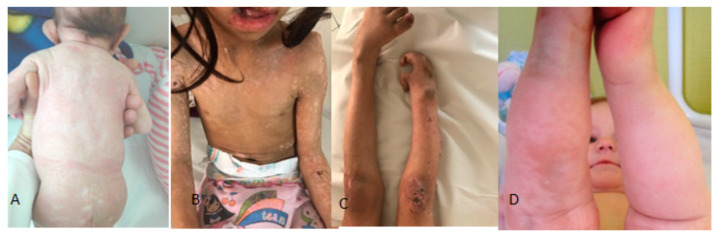
The clinical phenotype of the posterior aspect of the 6-month-old girl showed a unilaterally large and protruding left ear associated with spots of hypopigmentation all over the trunk. These skin stigmata have been evident since birth, which was followed by linear vesicular eruptions (**A**). The clinical phenotype of a 13-year-old girl showed multiple papillomas over the upper lip, around the mouth, and a cleft palate. Extensive dissemination of hypopigmented spots over the chest and hypoplasia of the nipples and the upper limbs (**B**). The phenotype of the lower limb of the same 13-year-old girl showed verrucoid papillomas over the right knee and a lobster claw on the right foot associated with limb length inequality (**C**). Unilateral congenital skin patches in a 20-month-old girl with GS over the right lower limb appear as hyperpigmented transverse lines (**D**).

**Figure 3:**
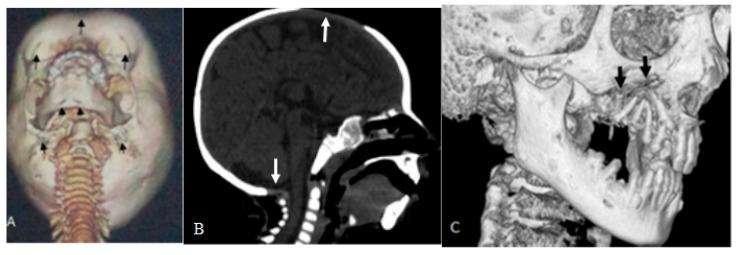
A 3D reconstruction of the craniofacial bones in extension (dynamic) in a 13-year-old girl with GS showed mid-line defective ossification of the metopic suture, causing bifid/bossing of the frontal area, bilateral bossing of the suprazygomatic arch, defective dentition of the lower jaw, several bony pits over the mandible, and the lambdoid area-arrow heads (**A**). A 3D sagittal reformatted CT scan of the skull of a 20-month-old girl with GS showed defective ossification of the cranial vault (arrow) and delayed closure of the posterior fontanelle (arrow) (**B**). A cranio-facial reconstruction CT scan of a 16-year-old girl with GS showed marked cystic cavitation of the temporal bone associated with excessive lytic changes of the upper jaw (arrows) and the mastoid, causing tremendous frailty of the mastoid bone. Note the severe dental dysplasia and oligodontia (**C**).

Skin manifestations are diverse but mostly distinctive. The skin lesions are often bilateral but asymmetrical over both lower limbs, initially red in color, patchy, and of different shapes and sizes. In addition, there are often areas of linear or reticular hyper- or hypopigmentation. Papillomas develop around the lips, gums, or the side of the nose. We describe the most striking skin features in three different unrelated children. The clinical phenotype of the posterior aspect of the 6-month-old girl showed a unilaterally large and protruding left ear associated with spots of hypopigmentation all over the trunk. These skin stigmata have been evident since birth, which was followed by linear vesicular eruptions (Figure 2A). The clinical phenotype of a 13-year-old girl showed multiple papillomas over the upper lip, around the mouth, and a cleft palate. Extensive dissemination of hypopigmented spots over the chest and hypoplasia of the nipples and the upper limbs (Figure 2B). The phenotype of the lower limb of the same 13-year-old girl showed verrucoid papillomas over the right knee and a lobster claw right foot associated with limb length inequality (Figure 3C). Unilateral congenital skin patches in a 20-month-old girl with GS over the right lower limb appear as hyperpigmented transverse lines.

### 3.3. Cranial Phenotype of Goltz Syndrome

Defective ossification and maldevelopment of the cranial and facial bones have been well encountered in our patients. A 3D reconstruction CT scan showed a constellation of unusual skull bone changes in three unrelated patients with GS. 3D reconstruction of the craniofacial bones in extension (dynamic) in a 13-year-old girl with GS showed mid-line defective ossification of the metopic suture, causing bifid/bossing of the frontal area, bilateral bossing of the suprazygomatic arches (arrows), defective dentition of the lower jaw, several bony pits over the mandible, and the lambdoid area-arrow heads (Figure 3A). A 3D sagittal reformatted CT scan of the skull in a 20-month-old girl with GS showed defective ossification of the cranial vault (arrow) and delayed closure of the posterior fontanelle (arrow) (Figure 3B). A cranio-facial reconstruction CT scan of a 16-year-old girl with GS showed marked cystic cavitation of the temporal bone associated with excessive lytic changes of the upper jaw (arrows) and the mastoid, causing tremendous frailty of the mastoid bone. Note the severe dental dysplasia and oligodontia (Figure 3C).

### 3.4. Craniocervical Junction in Goltz Syndrome

We demonstrate that the osseous evolution of the cranio-cervical and cervical spines in children of different ages with GS is diverse. Congenital and anatomical disruption of the osseous structures, which adversely affect the craniocervical complex, can result in a constellation of neurological deficits and can lead to life-threatening complications. The connection between alopecia, defective ossification of the cranium, aplasia of the odontoid process, and disruption in the development of the skull base associated with the craniocervical and cervical spines is well illustrated in two children with GS.

**Figure 4:**
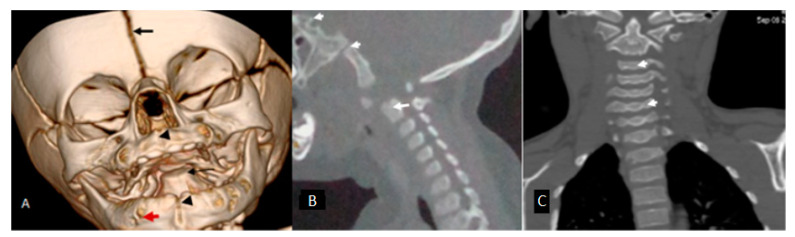
A 3D reconstruction CT scan of a 7-month-old girl with GS showed widely opened sutures, a short philtrum (pseudocleft lip), and midline clefting of the mandible (arrow heads). Agenesis of the odontoid process (arrow) and the multiple mandibular pits (red arrow) (**A**). A sagittal reformatted CT scan of a 20-month-old girl with GGS showed extensive anatomical disruption of the craniocervical junction, reflecting skull base synchondrosis—arrowhead. Dysplastic clivus, rudimentary atlas, and bifid dysplastic odontoid associated with dysplasia of the cervical vertebral bodies also note the thin and retarded closure of the posterior fontanelle-arrow (closure of the posterior fontanelle should take place after the age of 8 weeks of life) (**B**). A coronal reformatted CT scan of an 8-year-old girl with GS showed synchondrosis of C3-T1. The persistence of cervical spine and cervico-thoracic synchondrosis (arrow heads) can lead to progressive mal-alignment of the vertebral column, particularly in preadolescence (**C**).

A 3D reconstruction CT scan of a 7-month-old girl with GS showed widely opened sutures, a short philtrum (pseudo cleft lip), and midline clefting of the mandible (arrow heads). Agenesis of the odontoid process (arrow) Note the multiple mandibular pits (red arrow) (Figure 4A).

A sagittal reformatted CT scan of a 20-month-old girl with GS showed extensive anatomical disruption of the craniocervical junction, reflecting skull base synchondrosis—arrowhead. Dysplastic clivus, rudimentary atlas, and bifid dysplastic odontoid associated with dysplasia of the cervical vertebral bodies also note the thin and retarded closure of the posterior fontanelle-arrow (closure of the posterior fontanelle should take place after the age of 8 weeks of life) (Figure 4B). A coronal reformatted CT scan of an 8-year-old girl with GS showed synchondrosis of C3-T1. The persistence of the cervical spine and cervico-thoracic synchondrosis (arrow heads) can lead to progressive malalignment of the vertebral column, particularly in preadolescence (Figure 4C).

### 3.5. Limb Defects in Goltz Syndrome

**Figure 5:**
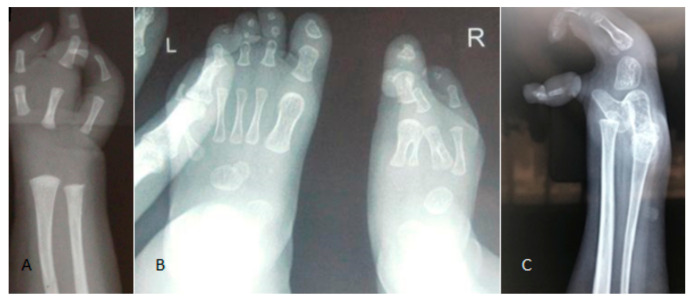
An AP hand radiograph of a six-month-old girl with GS showed a lobster claw hand with oligodactyly (aplasia of the second and fourth fingers with preservation of the thumb) and index and distal dysplasia of the fifth finger, associated with total aplasia of the carpal bones (**A**). An AP foot radiograph of a month-old girl showed a split foot deformity of the right foot (**B**). An AP foot radiograph of a 14-year-old girl with GS showed ectrodactyly (preservation of the big toe associated with tarsal and metatarsal agenesis) (**C**).

Upper and lower hand and foot defects have been well demonstrated in all children. The radiographic features of diverse forms of lobster claw hands, ectrodactly, and agenesis of some fingers and toes. An AP hand radiograph of a six-month-old girl with GS showed a lobster claw hand with oligodactyly (aplasia of the second and fourth fingers with preservation of the thumb) and index and distal dysplasia of the fifth finger associated with a total aplasia of the carpal bones (Figure 5A). An AP foot radiograph of a month-old girl showed a split foot deformity of the right foot (Figure 5B). An AP foot radiograph of a 14-year-old girl with GS showed ectrodactyly (preservation of the big toe associated with tarsal and metatarsal agenesis) (Figure 5C).

## 4. Discussion

Reviewing the academic literature in patients with Goltz syndrome showed specific interest in the traditional, well-known features.

There were restrictions on adding new data, and the only imaging studies carried out were either conventional radiographs or neuroimaging. These procedures constrained the depth of knowledge regarding the concomitant pathologies of the cranial, craniocervical, and upper spines. In accordance with our experience, we noticed that the vast majority of patients with ectodermal dysplasia are at high risk of unseen cranial, cranio-cervical, and spine malformations.

At birth, the anterior fontanel is present, and it is supposed to be completely closed maximally at the age of 18 months (though some variations can occur). Synchondroses seen in the neural arches, body, and odontoid process are supposed to fuse at the first few years of age [10]. Synchondrosis of the neurocentral junction of the spine has been identified as the etiology behind the development of adolescent scoliosis [11]. Through the detailed anatomical analysis of the cranial-axial via a reformatted CT scan, we proved that the occurrence of disrupted anatomical development occurred during the cartilaginous stage of embryological development of the spine.

Goltz syndrome is an ectodermal dysplasia characterized by dermatological lesions that are evident at birth [1,2,3]. The phenotype of the lesions is characterized by either linear streaks of skin hypoplasia or pigmentary alterations that follow the Blaschko line. Ectodermal dysplasias are single-gene heritable disorders with variable inheritance patterns encompassing most of the organs that stem from the ectoderm [12]. Patients with ectodermal dysplasia traditionally show a combination of sparse hair, disturbed sweating because of insufficient or absent sweat glands, and defective development of the skull bones [13].

There have been a remarkable number of published papers concerning the extent of the associated abnormalities in patients with focal dermal hypoplasia. Kore-Eda et al. [14] described multiple giant papillomas. Patel et al. [15] described a severe type of Goltz syndrome. Tail-like appendage in focal dermal hypoplasia (Goltz syndrome) [16]. Midline thoracoabdominal schisis and limb defects in an infant [17]. The occurrence of atypical facial clefting in a patient with Goltz syndrome [18]. A horseshoe kidney in a patient with Goltz syndrome [19]. Congenital ventral hernia in association with focal dermal hypoplasia syndrome [20]. A constellation of skin lesions, cranio-facial deformity, and limb deformity in five patients with Goltz syndrome [21]. Multiple squamous papillomas of the esophagus are associated with Goltz syndrome [22]. Osteochondroma of the humerus has been reported in association with focal dermal hypoplaisa [23]. Vertebral solid aneurysmal bone cyst variant [24]. Truncus arteriosus and other lethal internal anomalies in Goltz syndrome [25]. Intestinal malrotation and mediastinal dextroposition [26]. Giant cell tumor of bone [27]. Lee et al. described the classical features of Goltz syndrome [28]. Xu et al. described extensive blaschkoid macules and patches since birth in connection with Goltz syndrome [29]. Dusak et al. described several neurological deficits by means of neuroimaging studies in 17 patients with anhidrotic ectodermal dysplasia [30]. They suggested that anhidrotic ectodermal dysplasia can lead to a wide spectrum of neurological abnormalities, referring to the possible connection with ectodermal dysplasia. They did not perform CT scan studies on the cranium. None of the above-mentioned papers was able to further describe the craniofacial, craniocervical, or spine malformation complex as we performed in our series of patients.

We believe that the constellation of abnormalities in children with Goltz syndrome is not entirely due to genetics. Environmental factors also exist, as the clinical sessions that we organized with the mothers all confirmed that episodes of unexplained fever and hyperthermia were so evident in the first and second trimesters. Evidently, these mothers are the genetic carriers of ectodermal dysplasia, where the sweating apparatus is malfunctioning. Therefore, we suspect that the severity of the maternal hyperthermia might adversely affect the process of embryogenesis. Unfortunately, ultrasounds performed during gestation as arranged by the gynecologists made it difficult to discover the aforementioned fetal abnormalities because of the minimal awareness of gynecologists in connecting the maternal clinical phenotype with the expected deformities. The clinical phenotype of the mothers was unusual. They showed variable clinical features of sparse hair, faint eyebrows, abnormal dentition, and some persistence of deciduous teeth. Intolerance to hot weather was also a cardinal sign. Breastfeeding was a major complaint due to hypoplastic nipples and insufficient milk production. In the literature, several studies have described the correlation between the severity of fetal malformations and maternal hyperthermia in families with ectodermal dysplasia. The magnitude of the malformation is strongly correlated with the stage of embryonic and fetal development and the season of gestation [31,32].

We wish to stress that the recent application of reflectance confocal microscopy (RCM), which is a high-resolution, noninvasive imaging technique, has been successfully applied to help dermatologists in the diagnosis of certain skin lesions/tumors and to differentiate between melanoma and non-melanoma in patients with suspected and unusual skin pathologies, aid in the diagnosis, and rule out inflammatory skin lesions [33,34]. Furthermore, the value of a confocal microscope as a non-invasive technique is also applicable in studying skin biopsies. This can be done through the morphological recognition of the sweat glands and sweat ducts in the palms of patients with ectodermal dysplasia [35].

## 5. Conclusions

Ectodermal dysplasia forms extensive clinical and genetic categories with variable and diverse phenotypic/genotypic characterizations, as well as misleading etiological heterogeneity. The importance of early diagnosis of abnormal development of the associated craniocervical and axial components is empirical. Particularly when reconstruction surgery for a cleft palate or ectrodactyly is scheduled, it is quite difficult to plan a preoperative strategy for major operations without proper recognition of the associated defects.

## Data Availability

Data is unavailable due to ethical restrictions.
